# A Pan-Cancer Analysis of SLC12A5 Reveals Its Correlations with Tumor Immunity

**DOI:** 10.1155/2021/3062606

**Published:** 2021-09-29

**Authors:** Yi Jiang, Hong-li Liao, Li-ya Chen

**Affiliations:** Department of Pathology, Wenzhou Central Hospital, Wenzhou, Zhejiang, China

## Abstract

**Background:**

Solute carrier family 12 member 5 (SLC12A5) has been reported to play an oncogenic role in certain malignancies. Its prognostic roles and immune mechanisms of action in human cancers, however, remain largely unknown.

**Methods:**

Data derived from TCGA, GEPIA, and TIMER databases were utilized to delve into the expressing patterns, prognostic values, clinical significances, and tumor immunity of SLC12A5 in tumors. Additionally, the association of SLC12A5 expressions with tumor mutation burden (TMB), methyltransferases, and mismatch repairs (MMRs) was also analyzed.

**Results:**

Herein, we observed that SLC12A5 was significantly overexpressed in various malignancies, and SLC12A5 levels correlated with overall survival, disease-specific survival, and tumor stage of certain cancers. Furthermore, we noticed that SLC12A5 was distinctly associated with methyltransferases, mismatch repair proteins, TMB, and MSI in human cancers.

**Conclusions:**

SLC12A5 may act as a potential prognostic and immunological biomarker and therapeutic target for human cancers.

## 1. Introduction

Cancer is one of the greatest threats to human health. Many types of tumors have complex clinical and pathologic characteristics, and the extremely complex pathogenesis of tumors has yet to be explored [1, 2]. In recent years, the tumor microenvironment is regarded as an essential participant in tumor progression [3]. In turn, cancer progression results in an immunosuppressive tumor microenvironment [4]. Cancers and its microenvironment are closely related and constantly interacted, influencing the initiation and progression of human cancers [5, 6]. As the important component of the tumor microenvironment, immune cells have been reported to play crucial roles not only in immune modulation but also in cancer progression [7, 8]. Increasing evidence shows that immune-related mechanisms play essential roles in the tumorigenicity and progressions of human tumors, and immunotherapy has been considered as a novel direction in clinical treatments of tumors [9, 10]. The occurrence of immune checkpoint inhibitors (ICIs) has resulted in the reformation of the current status for patients with advanced malignancies. For instance, CTLA4 inhibitors, PD-L1, and PD-1 have exhibited superior efficacy in malignant melanoma and lung tumor [11, 12]. Additionally, various new types of immune checkpoints such as V-domain Ig suppressor of T cell activation, A virus cellular receptor 2, CD276 molecule, and lymphocyte activating 3 are gradually being discovered [13, 14]. Unfortunately, only a small portion of certain cancer patients responds positively to immunotherapy [15, 16]. Thus, it is urgently necessary to explore other targets.

Solute carrier (SLC) family is the largest family of transmembrane transport proteins, including 65 families with over 400 transporter genes, which could transport various substances such as nutrients, ions, metabolites, and drugs across the cell membranes [17, 18]. Solute carrier family 12 member 5 (SLC12A5) encodes K^+^-Cl^−^ cotransporter 2, which is related to various central and peripheral nervous system diseases. It has been reported that the loss of function of SLC12A5 was significantly associated with neurological disorders, such as epilepsy, autism, and schizophrenia [19–21]. In addition, studies have found that SLC12A5 played a vital role in the regulation of insulin secretion [22]. For human cancers, the expression and function of SLC12A5 were rarely reported. In bladder cancer, SLC12A5 was shown to be highly expressed and its overexpression promoted the proliferation and metastasis of tumor cells via increasing SOX18 expression [23]. The prognostic value of SLC12A5 was also reported in ovarian carcinoma and colorectal cancer [24, 25]. However, similar studies of SLC12A5 in other cancer types remained in infancy.

In this research, we comprehensively analyzed the expressing patterns, prognostic values, and clinical significances of SLC12A5 in pan-cancers. What is more, we focused on the association between SLC12A5 expression and six tumor-infiltrating immune cells (TIICs) and immunosuppressive molecules in pan-cancers. Taken together, our work revealed that SLC12A5 might serve as a biomarker indicating tumor progression and prognosis and play multifaceted roles in modulating tumor immunity.

## 2. Materials and Methods

### 2.1. Data Collection

RNA sequencing and clinical data of all samples were downloaded from The Cancer Genome Atlas (TCGA) database (11069 samples from 33 types of cancer) through the UCSC Xena (https://xena.ucsc.edu/). To extract the transcriptional expression data of SLC12A5 from the downloaded data sets, Strawberry Perl (Version 5.32.0, http://strawberryperl.com/) was employed. We then conducted Wilcoxon signed-rank test to estimate differential SLC12A5 expressions between the normal and tumor groups. All expression data were normalized by log2 (TPM + 1) transformation.

### 2.2. Gene Expression Profiling Interactive Analysis (GEPIA)

GEPIA (http://gepia.cancer-pku.cn/) is a newly developed interactive web server for analyzing TCGA and GTEx projects. In the current study, the GEPIA database was used to assess the expression patterns of SLC12A5 in the normal and tumor groups.

### 2.3. Survival Analysis of SLC12A5 in Pan-Cancer

The overall survival and disease-specific survival of SLC12A5 in pan-cancer were evaluated using the Cox regression analysis. Hazard ratio (HR) value greater than one means that SLC12A5 is a risk factor in cancer; in contrast, HR value less than one represents that SLC12A5 is a protection factor in cancer. What is more, the Kaplan-Meier methods were utilized to estimate the difference between the high and low expressing groups based on the best separation of SLC12A5 expression.

### 2.4. Associations of SLC12A5 Expression with Clinical Stage of Pan-Cancer

To assess clinical significance of SLC12A5 in pan-cancers, clinical stage data were extracted using TCGA database. Then, we conducted the Wilcoxon signed-rank test or Kruskal-Wallis test to study the relationships between SLC12A5 expression and clinical stage of patients.

### 2.5. TIMER Database

TIMER (https://cistrome.shinyapps.io/timer/) is a web server for the comprehensive analysis of TIICs [26]. We used the “Gene” module to assess the relationships between SLC12A5 expressions and six immune cell infiltration levels (dendritic cells, macrophages, neutrophils, CD8+ T cells, CD4+ T cells, and B cells).

### 2.6. Mismatch Repairs (MMRs), Microsatellite Instability (MSI), and Tumor Mutational Burden (TMB) of SLC12A5 in Various Cancers

TMB and MSI are regarded as important factors impacting the initial and progression of human tumors. Emerging evidence discovered that methyltransferase dysregulation is significantly associated with a variety of cancers, which make some of them viable targets for tumor treatment strategies [27, 28]. Additionally, studies have demonstrated that cancers with a great many somatic mutations may be susceptible to immune checkpoint blockade [29, 30]. In the study, we assess the relationship between SLC12A5 expression with TMB, MSI, methyltransferases, and MMRs.

### 2.7. Statistical Analysis

The Wilcoxon signed-rank tests were applied to compare the expressions of SLC12A5 in cancer specimens with those in nontumor specimens. The Kaplan-Meier method with log-rank test and Cox analysis were utilized to estimate the effects of SLC12A5 on OS and DSS of patients. The Kruskal-Wallis tests were used to explore the association between the clinical stage and SLC12A5 expression. *P* < 0.05 was considered to be statistically significant. R software (version 3.6.1) was used for statistical analyses, and the R packages used in each step are mentioned above.

## 3. Results

### 3.1. The Expression Patterns of SLC12A5 in Pan-Cancer

**Figure 1:**
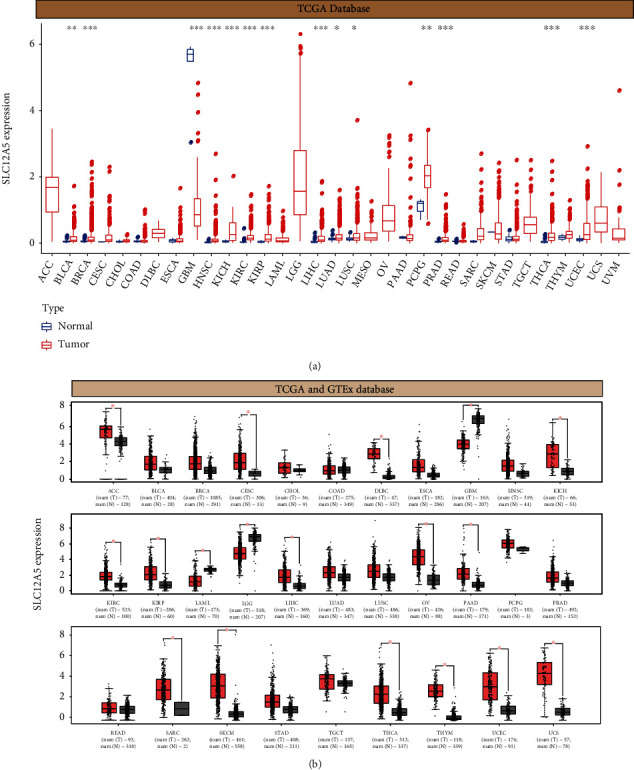
SLC12A5 expression levels in different cancer types. (a) Human SLC12A5 levels across different cancer types from TCGA database. (b) The dysregulated expressions of SLC12A5in tumor samples across different cancer types in the GEPIA database. ^∗^*P* < 0.05, ^∗∗^*P* < 0.01, and ^∗∗∗^*P* < 0.001.

Using the SLC12A5 expression data for 33 cancers retrieved from TCGA database, our group observed that SLC12A5 was overexpressed in various types of tumors, including BLCA, BRCA, HNSC, KICH, KIRC, KIRP, LIHC, LUAD, LUSC, PCPG, PRAD, THCA, and UCEC tissues compared to their corresponding normal tissues. However, decreased SLC12A5 expression was found in GBM (*P* < 0.001) (Figure 1(a)). Given the lack of normal controls for some cancers in TCGA database, we used the GEPIA database containing data from both TCGA and GTEx databases to further explore the SLC12A5 expression status in pan-cancers, and as revealed in Figure 1(b), SLC12A5 was highly expressed in ACC, PAAD, CESC, DLBC, KICH, LAML, LIHC, SARC, THCA, KIRC, THYM, UCEC, KIRP, OV, and USC tissues compared with nontumor tissues, while downregulation of SLC12A5 was found in GBM and LGG (all *P* value < 0.05) compared with their corresponding adjacent noncancerous tissues.

### 3.2. The Association of SLC12A5 Expression with Prognosis and Tumor Stage of Human Cancers

**Figure 2:**
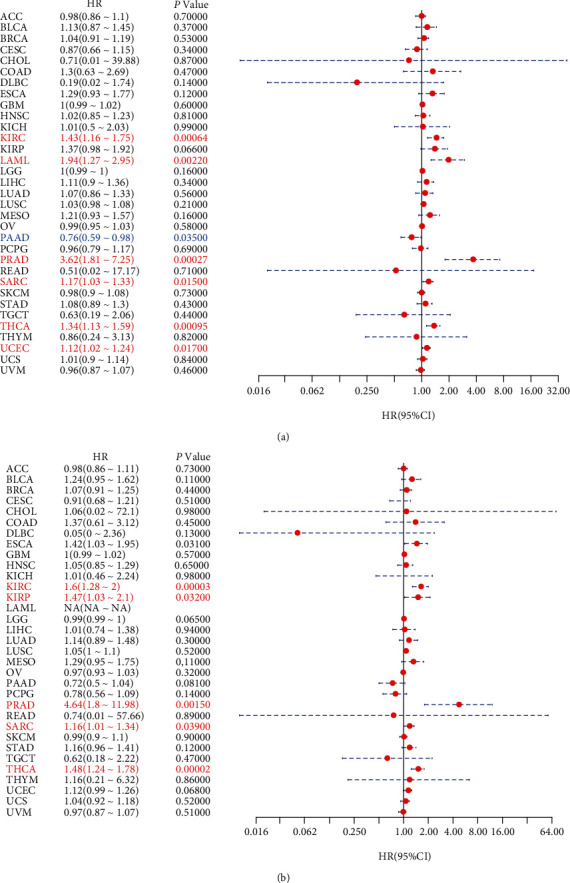
The effects of SLC12A5 on the outcome of various cancers. The effects of SLC12A5 on (a) overall survival and (b) disease-specific survival in 33 types of cancers.

**Figure 3:**
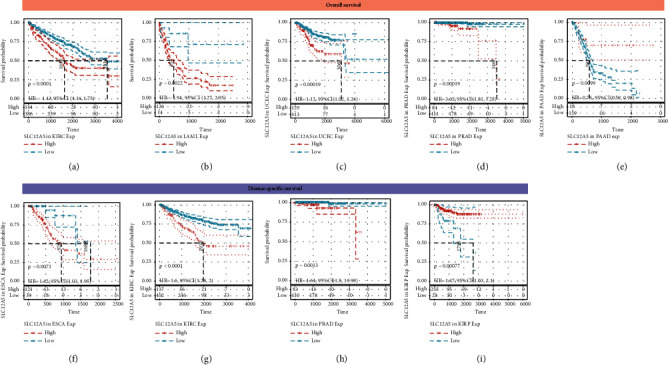
The survival curve of SLC12A5 in various tumors using the Kaplan-Meier methods. The survival curves of SLC12A5 for overall survival (OS) in (a) KIRC, (b) LAML, (c) UCEC, (d) PRAD, and (e) PAAD. The survival curve of SLC12A5 for disease-specific survival (DSS) in (f) ESCA, (g) KIRC, (h) PRAD, and (i) KIRP.

**Figure 4:**
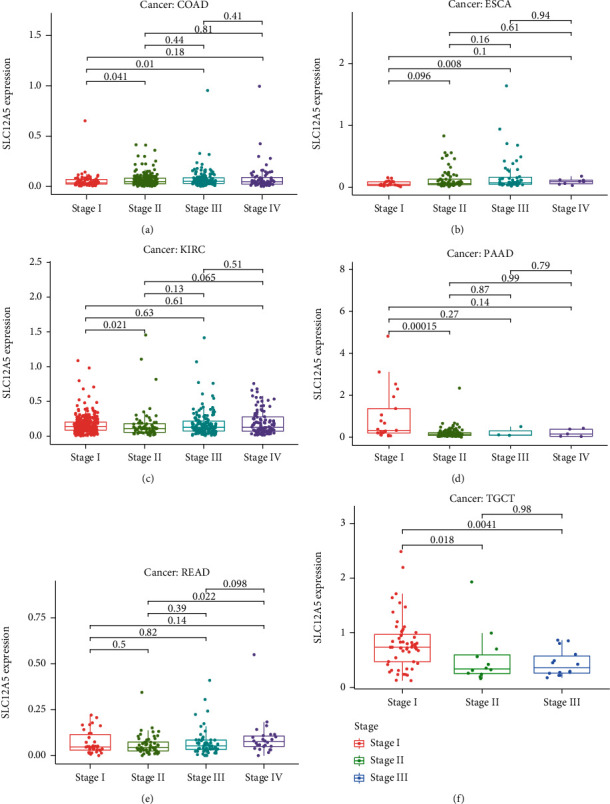
The expression of SLC12A5in tumor specimens with different stages in (a) COAD, (b) ESCA, (c) KIRC, (d) PAAD, (e) READ, and (f) TGCT.

We further explored the prognostic values of SLC12A5 in human cancers using the Cox analysis and Kaplan-Meier survival method. As illustrated in Figure 2(a), SLC12A5 expression was associated with OS in KIRC, LAML, PAAD, PRAD, SARC, THCA, and UCEC (all *P* < 0.05). Additionally, as shown in Figure 2(b), SLC12A5 expression was associated with DSS in KIRC, KIRP, PRAD, SARC, and THCA (all *P* < 0.05). Then, the Kaplan-Meier survival methods were further utilized to assess the prognostic impact of SLC12A5 in human cancers, and as revealed in Figures 3(a)–3(d), high SLC12A5 expression was distinctly associated with shorter overall survival of patients with KIRC, LAML, UCEC, and PRAD, whereas the lower the SLC12A5 expression level, the worse the OS of PAAD (Figure 3(e)). Furthermore, the results of the Kaplan-Meier curves indicated that increased expressions of SLC12A5 were associated with poor DSS in ESCA, KIRC, and PRAD (Figures 3(f)–3(h)), while with a favorable DSS in KIRP (Figure 3(i)). Moreover, using the Kruskal-Wallis test, we explore the expression levels of SLCA5 according to the tumor stage of human cancer patients. As displayed in Figures 4(a)–4(c), SLC12A5 had higher expression in advanced tumor stages of COAD, ESCA, and KIRC, while the lower the expression level of SLC12A5, the more advanced stage of PAAD, READ, and TGCT (Figures 4(d)–4(f)).

### 3.3. Association of SLC12A5 Expressions with TIICs and Immunosuppressive Molecules

**Figure 5:**
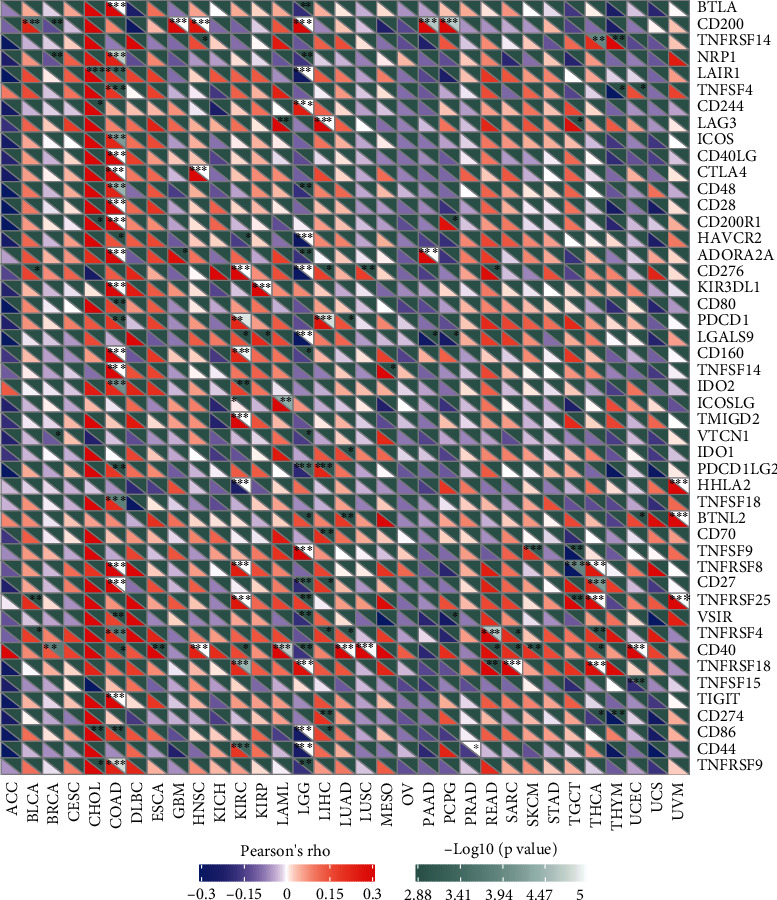
The associations between SLC12A5 expressions and pan-cancer immune checkpoint genes.

TIICs were a part of the tumor microenvironment that modulates cancer development and progression. Whether SLC12A5 affected immune infiltration had not been clarified. By the use of the TIMER database, we firstly estimated the association between SLC12A5 expressions and the infiltration of immune cells. We noticed that SLC12A5 expression was distinctly correlated with the level of immune infiltration of B cells in 15 types of cancers, CD8+ T cells in 10 types of cancers, CD4+ T cells in 22 types of cancers, and macrophages in 16 types of cancers (Supplementary Figure S1 and S2). Immunosuppressive molecules, such as immune checkpoints, could result in an immunosuppressive environment that allows tumor cells to escape antitumor immunity. In the current work, we also evaluated the relationship between SLC12A5 and immunosuppressive molecules. As revealed in Figure 5, SLC12A5 was significantly associated with three immunosuppressive molecules in BLCA, four in BRCA, five in CHOL, 28 in COAD, three in ESCA, two in GBM, four in HNSC, 12 in KIRC, two in KIRP, three in LAML, 23 in LGG, nine in LIHC, six in LUAD, two in LUSC, one in MESO, three in PAAD, four in PCPG, one in PRAD, four in READ, three in SARC, two in SKCM, four in TCGC, eight in THCA, three in THYM, four in UCEC, and three in UVM. Our findings suggested an important association between SLC12A5 and immunosuppressive molecules.

### 3.4. SLC12A5 Expressions, TMB, MSI, Methyltransferases, and MMRs

**Figure 6:**
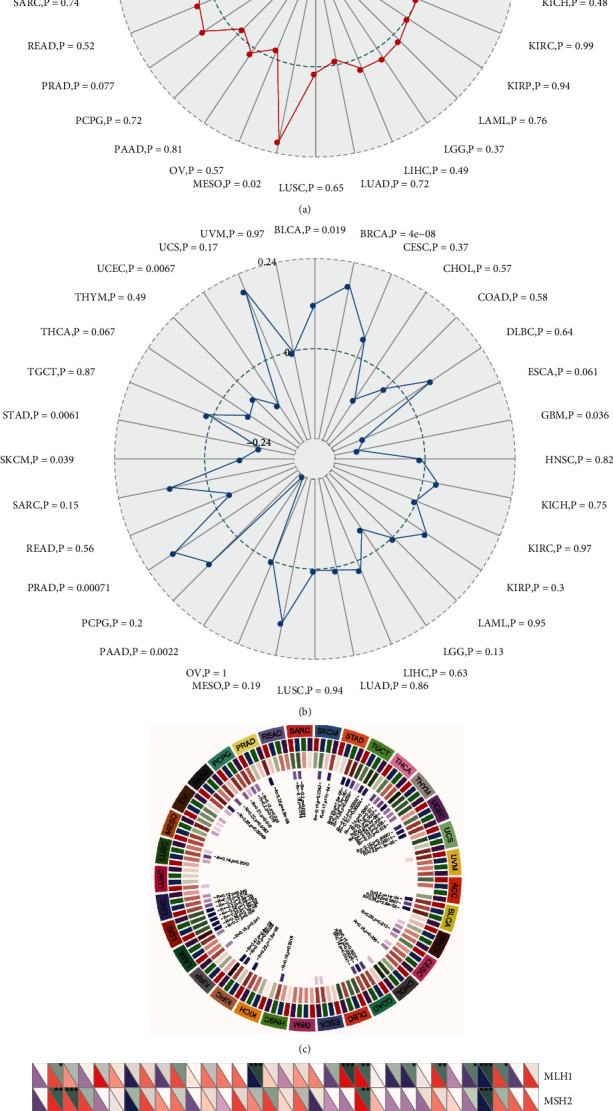
The correlations between SLC12A5 expression and (a) TMB, (b) MSI, (c) methyltransferases (DNMT1: red; DNMT2: blue; DNMT3A: green; DNMT3B: purple), and (d) mismatch repairs (MMRs) in various tumors. ^∗^*P* < 0.05; ^∗∗^*P* < 0.01; ^∗∗∗^*P* < 0.001.

Considering the role of TMB, MSI, methyltransferases, and MMRs in tumor progression, we firstly evaluated the association of SLC12A5 expression with them. As illustrated in Figure 6(a), SLC12A5 expressions exhibited distinctly positive associations with TMB in BRCA, MESO, and THCA, while it has negative correlations in STAD, TGCT, UCEC, and UVM. Additionally, we found that high levels of SLC12A5 were considerably positive with MSI in PRAD, BRCA, and BLCA, but significantly negatively in GBM, PAAD, SKCM, STAD, and UCEC (Figure 6(b)). Then, we assess the association between four methyltransferases and SLC12A5 expression. We observed that SLC12A5 expressions were associated with one or more methyltransferases in 21 tumor types (Figure 6(c)). Besides, SLC12A5 expression was significant with MLH1 in nine tumor types, with MSH2 in four cancers, with MSH6 in five tumors, with PMS2 in six tumor types, and with EPCAM in four cancer types (Figure 6(d)).

## 4. Discussion

Herein, we comprehensively and systematically explored the roles of SLC12A5 in 33 human cancers. Firstly, our results suggested that SLC12A5 was highly expressed in UCEC, KIRC, PAAD, ACC, CESC, DLBC, KICH, LAML, LIHC, KIRP, SARC, THCA, THYM, OV, and USC tissues compared with nontumor tissues, while it only decreased in GBM and LGG compared to their corresponding normal controls, indicating that SLC12A5 might act as a tumor promoter in human cancers. Secondly, combining with the results of the Cox analysis and Kaplan-Meier method, we found that high SLC12A5 expression correlated with worse overall survival of KIRC, LAML, PRAD, and UCEC, while it correlated with favorable overall survival of PAAD. Additionally, elevated expressions of SLC12A5 predicted a poor disease-specific survival in KIRC and PRAD. The results showed the potential for SLC12A5 as a prognostic biomarker in certain malignancies. Then, we investigated the clinical significances of SLC12A5 in pan-cancers, and we noticed that SLC12A5 was higher in advanced tumor stages of COAD, ESCA, and KIRC, while it was lower in advanced tumor stages of PAAD, READ, and TGCT.

As a hub component of the tumor microenvironment, tumor immune infiltrating cells acted as a potential regulator in the progression of various tumors [31]. An interesting result in the current study was that SLC12A5 expression was significantly associated with various immune infiltration levels in human cancers, especially in PRAD, LUSC, LUAD, LGG, KIRP, KIRC, HNSC, CHOL, and BRCA, suggesting that SLC12A5 may mediate cancer progression by affecting the immune infiltrate in malignancies. Immunosuppressive molecules exhibited a regulatory effect on the tumor microenvironment. Interestingly, we observed that SLC12A5 was remarkably correlated with immunosuppressive molecules, especially in COAD, KIRC, and LGG. Concretely, in COAD and KIRC, SLC12A5 had a positive relationship with several immunosuppressive molecules, such as PDCD1, CD160, TNFRSF8, CD40, and IDO2. However, a negative association between SLC12A5 expressions and most immunosuppressive molecules was found in LGG. The results reflected the different regulatory relationships between SLC12A5 and immunosuppressive molecules in different cancers.

TMB and MSI are frequently observed in human cancers and can serve as the predicting factors for cancer treatment efficacy. We found that SLC12A5 expression had positive correlations with TMB in BRCA, MESO, and THCA, while it had negative correlations in STAD, TGCT, UCEC, and UVM. Additionally, high SLC12A5 expression was considerably positively correlated with MSI in BLCA, BRCA, and PRAD, but significantly negatively correlated in GBM, PAAD, SKCM, STAD, and UCEC. Methyltransferase is a well-characterized epigenetic hallmark in malignancies, and several methyltransferases are now validated therapeutic targets [32, 33]. Additionally, it is reported that tumors with mismatch repair protein defects may be more susceptible to immune checkpoint blockade [34, 35]. In this study, we estimated the association of SLC12A5 with methyltransferases and mismatch repair proteins, finding that SLC12A5 expression was distinctly associated with the methyltransferases and mismatch repair proteins of several tumors.

Collectively, we uncover that SLC12A5 is remarkably associated with prognosis and progression of human cancers. Importantly, robust associations of SLC12A5 with tumor immunity are found in the current study. Nevertheless, some limitations should not be ignored. Due to the limited clinical information, some selection biases are inevitable. Additional research with larger sample size is required to verify and complement our findings. Additionally, the compelling evidences of SLC12A5 protein levels in human cancers are insufficient. Another limitation of this study is that the exact association between SLC12A5 and tumor immunity remains to be elucidated. More studies are needed to draw definitive conclusions in the future.

## Data Availability

The authors certify that all the original data in this research could be obtained from public database. All data generated or analyzed during this study are included in this article.

## Supplementary Materials

Supplementary 1 Supplementary Figure S1: correlations of SLC12A5 expressions with immune infiltration level in, from top to bottom, BRCA, CHOL, HNSC, KIRC, KIRP, PRAD, LUSC, LUAD, LIHC, and LGG.

Supplementary 2 Supplementary Figure S2: correlation of SLC12A5 expression with immune infiltration level in various tumors.

## References

[1] Siegel R. L., Miller K. D., Jemal A. (2020). Cancer statistics, 2020. *CA: a Cancer Journal for Clinicians*.

[2] Mullard A. (2020). Addressing cancer’s grand challenges. *Nature Reviews Drug Discovery*.

[3] Hinshaw D. C., Shevde L. A. (2019). The tumor microenvironment innately modulates cancer progression. *Cancer Research*.

[4] Quail D. F., Joyce J. A. (2013). Microenvironmental regulation of tumor progression and metastasis. *Nature Medicine*.

[5] Pitt J. M., Marabelle A., Eggermont A., Soria J. C., Kroemer G., Zitvogel L. (2016). Targeting the tumor microenvironment: removing obstruction to anticancer immune responses and immunotherapy. *Annals of Oncology*.

[6] Wu T., Dai Y. (2017). Tumor microenvironment and therapeutic response. *Cancer Letters*.

[7] Fujii S. I., Shimizu K. (2019). Immune networks and therapeutic targeting of iNKT cells in cancer. *Trends in Immunology*.

[8] Sabado R. L., Balan S., Bhardwaj N. (2017). Dendritic cell-based immunotherapy. *Cell Research*.

[9] Morrison A. H., Byrne K. T., Vonderheide R. H. (2018). Immunotherapy and prevention of pancreatic cancer. *Trends Cancer*.

[10] O’Donnell J. S., Teng M. W. L., Smyth M. J. (2019). Cancer immunoediting and resistance to T cell-based immunotherapy. *Nature Reviews Clinical Oncology*.

[11] Furue M., Ito T., Wada N., Wada M., Kadono T., Uchi H. (2018). Melanoma and immune checkpoint inhibitors. *Current Oncology Reports*.

[12] Nabet B. Y., Esfahani M. S., Moding E. J. (2020). Noninvasive early identification of therapeutic benefit from immune checkpoint inhibition. *Cell*.

[13] Qin S., Xu L., Yi M., Yu S., Wu K., Luo S. (2019). Novel immune checkpoint targets: moving beyond PD-1 and CTLA-4. *Molecular Cancer*.

[14] Havel J. J., Chowell D., Chan T. A. (2019). The evolving landscape of biomarkers for checkpoint inhibitor immunotherapy. *Nature Reviews Cancer*.

[15] Hegde P. S., Chen D. S. (2020). Top 10 challenges in cancer immunotherapy. *Immunity*.

[16] Martin J. D., Cabral H., Stylianopoulos T., Jain R. K. (2020). Improving cancer immunotherapy using nanomedicines: progress, opportunities and challenges. *Nature Reviews Clinical Oncology*.

[17] Liu X. (2019). SLC family transporters. *Advances in Experimental Medicine and Biology*.

[18] César-Razquin A., Snijder B., Frappier-Brinton T. (2015). A call for systematic research on solute carriers. *Cell*.

[19] Fukuda A., Watanabe M. (2019). Pathogenic potential of human *SLC12A5* variants causing KCC2 dysfunction. *Brain Research*.

[20] Kahle K. T., Khanna A. R., Duan J., Staley K. J., Delpire E., Poduri A. (2016). The KCC2 cotransporter and human Epilepsy. *The Neuroscientist*.

[21] Merner N. D., Chandler M. R., Bourassa C. (2015). Regulatory domain or CpG site variation in SLC12A5, encoding the chloride transporter KCC2, in human autism and schizophrenia. *Frontiers in Cellular Neuroscience*.

[22] Kursan S., McMillen T. S., Beesetty P. (2017). The neuronal K^+^Cl^−^ co- transporter 2 (*Slc12a5*) modulates insulin secretion. *Scientific Reports*.

[23] Wang L., Zhang Q., Wu P. (2020). SLC12A5 interacts and enhances SOX18 activity to promote bladder urothelial carcinoma progression via upregulating MMP7. *Cancer Science*.

[24] Yang G. P., He W. P., Tan J. F. (2019). Overexpression of SLC12A5 is associated with tumor progression and poor survival in ovarian carcinoma. *International Journal of Gynecological Cancer*.

[25] Xu L., Li X., Cai M. (2016). Increased expression ofSolute carrier family 12 member 5via gene amplification contributes to tumour progression and metastasis and associates with poor survival in colorectal cancer. *Gut*.

[26] Li T., Fan J., Wang B. (2017). TIMER: a web server for comprehensive analysis of tumor-infiltrating Immune Cells. *Cancer Research*.

[27] Hamamoto R., Nakamura Y. (2016). Dysregulation of protein methyltransferases in human cancer: an emerging target class for anticancer therapy. *Cancer Science*.

[28] Xu T. H., Liu M., Zhou X. E. (2020). Structure of nucleosome-bound DNA methyltransferases DNMT3A and DNMT3B. *Nature*.

[29] Tate J. G., Bamford S., Jubb H. C. (2019). COSMIC: the catalogue of somatic mutations in cancer. *Nucleic Acids Research*.

[30] Giacomelli A. O., Yang X., Lintner R. E. (2018). Mutational processes shape the landscape of _TP53_ mutations in human cancer. *Nature Genetics*.

[31] Gajewski T. F., Schreiber H., Fu Y. X. (2013). Innate and adaptive immune cells in the tumor microenvironment. *Nature Immunology*.

[32] Chang S., Yim S., Park H. (2019). The cancer driver genes *IDH1/2, JARID1C/ KDM5C* , and *UTX/ KDM6A*: crosstalk between histone demethylation and hypoxic reprogramming in cancer metabolism. *Experimental & Molecular Medicine*.

[33] Zou Z., Zhou S., Liang G. (2021). The pan-cancer analysis of the two types of uterine cancer uncovered clinical and prognostic associations with m6A RNA methylation regulators. *Molecular Omics*.

[34] Le D. T., Uram J. N., Wang H. (2015). PD-1 blockade in tumors with mismatch-repair deficiency. *The New England Journal of Medicine*.

[35] André T., Shiu K. K., Kim T. W. (2020). Pembrolizumab in microsatellite-instability-high advanced colorectal cancer. *The New England Journal of Medicine*.

