# Supplementary figures and images for: A Pan-Cancer Analysis of SLC12A5 Reveals Its Correlations with Tumor Immunity
Source: Dis Markers. 2021 Sep 29;2021:3062606. doi: 10.1155/2021/3062606 (PMC8495467; doi:10.1155/2021/3062606)

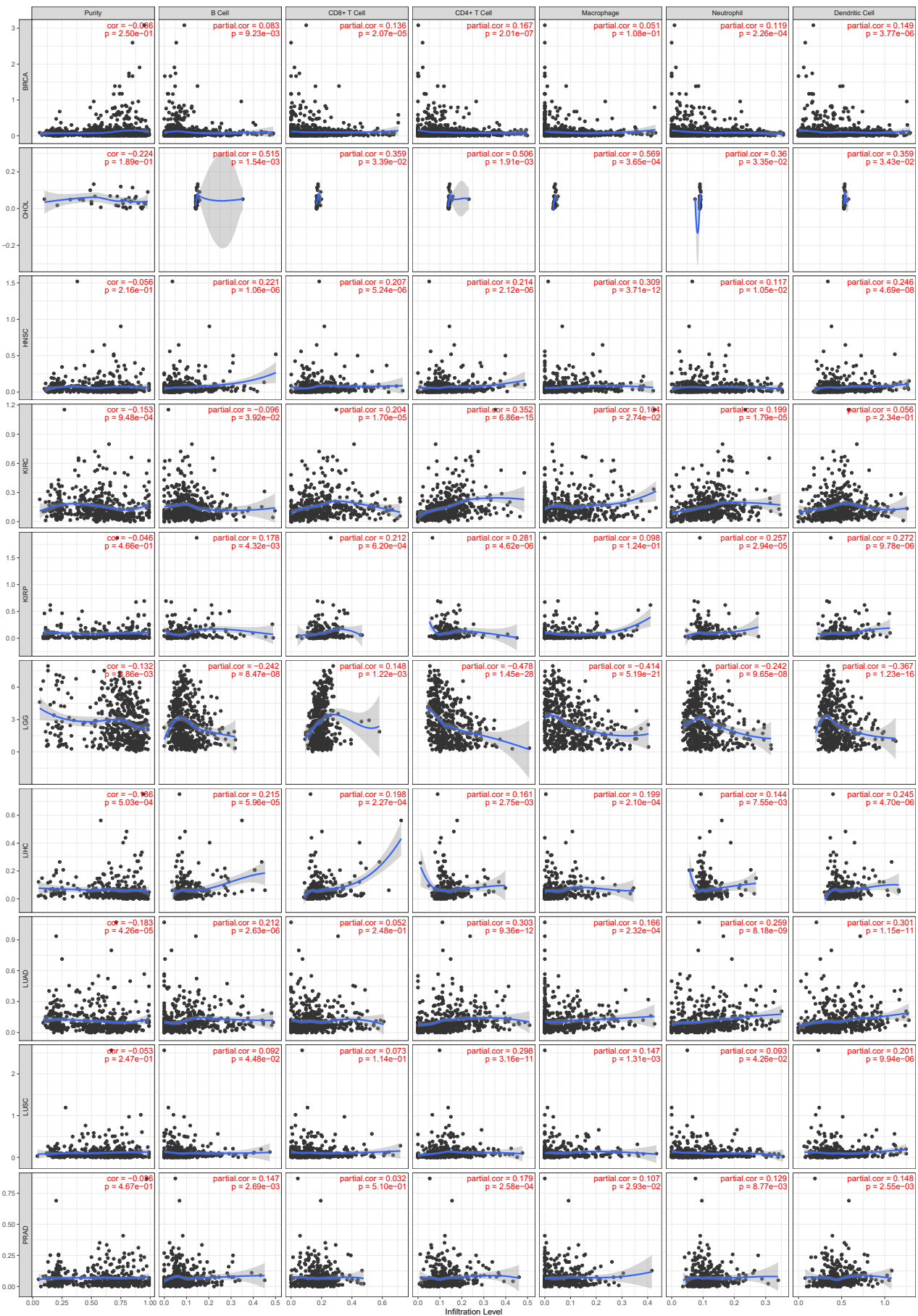

Supplement: Supplementary 1 — Supplementary Figure S1: correlations of SLC12A5 expressions with immune infiltration level in, from top to bottom, BRCA, CHOL, HNSC, KIRC, KIRP, PRAD, LUSC, LUAD, LIHC, and LGG. [file 3062606.f1.pdf]

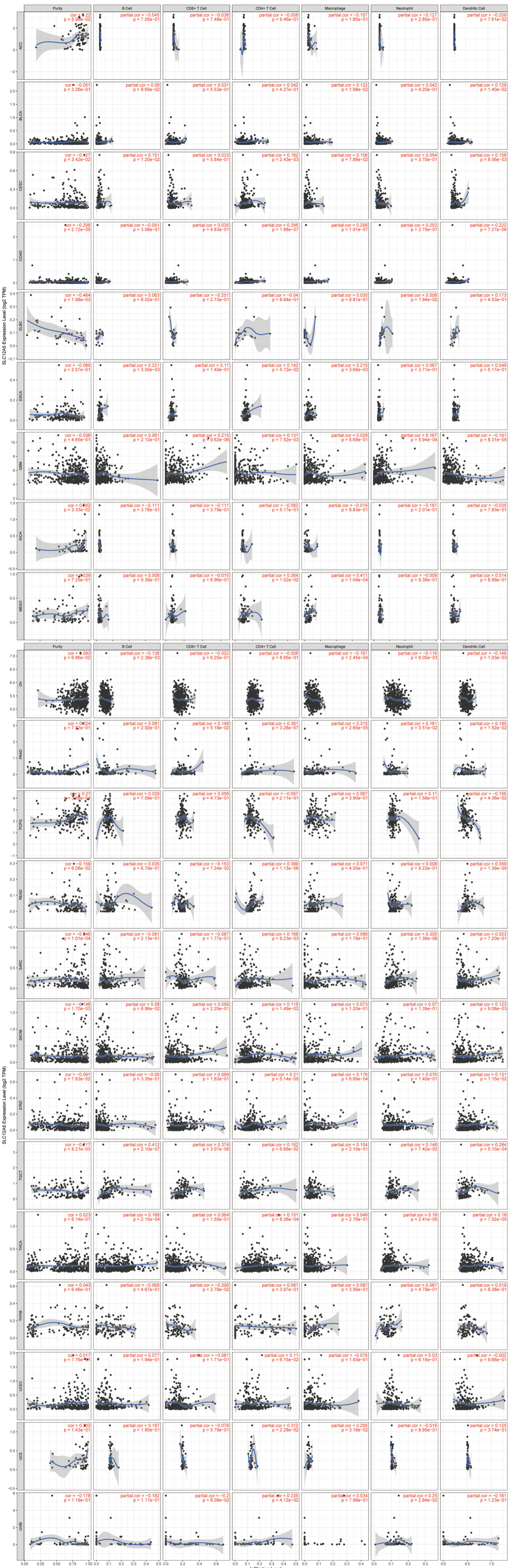

Supplement: Supplementary 2 — Supplementary Figure S2: correlation of SLC12A5 expression with immune infiltration level in various tumors. [file 3062606.f2.pdf]
